# Lifestyle and Chronic Comorbidity in Relation to Healthy Ageing in Community-Dwelling People Aged 80 and over: Preliminary Study from a Primary Health Care Service in Southern Spain

**DOI:** 10.3390/healthcare14020189

**Published:** 2026-01-12

**Authors:** Alberto Jesús García-Zayas, María del Carmen Márquez-Tejero, Juan Luis González-Caballero, Carmen Gómez-Gómez

**Affiliations:** 1 Department of Biochemistry and Molecular Biology, School of Medicine, University of Cadiz, 11002 Cádiz, Spain; 2 Unidad de Gestión Clínica Las Delicias, Distrito de Atención Primaria Jerez Costa Noroeste, 11406 Jerez de la Frontera, Spain; 3 Department of Statistics and Operations Research, Faculty of Medicine, University of Cadiz, 11002 Cádiz, Spain

**Keywords:** healthy ageing, daily autonomy, cognitive function, chronic comorbidities, oldest adults, primary care, blood biochemical biomarkers, Mediterranean diet, lifestyle, latent profile analysis

## Abstract

**Background/Objectives**: Healthy ageing, focused on maintaining daily autonomy and cognitive function despite chronic comorbidities, poses a challenge for public health systems, especially for those aged ≥80, given the expected increase in this population. Promoting a healthy lifestyle in this group is essential to achieving this goal, with primary care services playing a key role in this effort. Therefore, our objective was to profile the participants based on these characteristics. **Methods:** The study included 222 non-institutionalized, dementia-free individuals (mean age 84.58 ± 3.72 years, 56.3% women) recruited from a primary healthcare service. Data were collected from medical records and interviews, including the cognitive Pfeiffer test, the functional Barthel index (BI), and ad hoc questionnaires (for lifestyle variables). Latent profiling analysis (LPA) was used to classify the participants. **Results**: The participants reported social support (97.7%), low-risk alcohol consumption (94.6%), adherence to the Mediterranean diet (85.1%), physical activity (74.8%), and never smoking (72.5%). Hypertension (86.5%), cataracts (74.3%), and osteoarticular diseases (68.5%) were the most frequent chronic conditions. Women showed a significantly different distribution of certain variables and a higher number of comorbidities (6.34 ± 2.38) than men (5.58 ± 2.44) (*p* = 0.019). After LPA, we found that 38.29% of individuals met characteristics compatible with healthy ageing, predominantly male (60%); the association of a high probability of cognitive impairment with a high degree (severe or total), exhibited by the profiles likely >85% women (18.5% of individuals); physical activity, smoking, osteoporosis, anxiety, COPD, chronic kidney disease (CKD), and creatinine blood levels exhibited statistical differences between profiles; and the probability of dependence severity was associated with an increase in age, although cognitive status conservation was associated being male. **Conclusions:** The studied +80 group seems to follow a healthy lifestyle, as self-reported. Women fare worse than men in resilient ageing. While common factors related to dysfunctionality did not differentiate between profiles, CKD, an increasingly common age-related condition, did.

## 1. Introduction

Nowadays, people in developed countries are living longer and in better health, so the meaning of “elderly” is being redefined and reclassified, and old age is now considered to be 75 years or over [1]. On the other hand, it has been observed that it is the cognitive status, or a *cognitive clock*, that marks the stages, proposing a classification based on cognition, which remains stable until about 80, then declines moderately until 90, and then deteriorates precipitously [2]. However, chronological age cannot be ignored, as it remains the main risk factor for dementia, disability, and chronic comorbidity, which are all highly prevalent during this stage of life. Various studies agree on the most common chronic conditions in older adults: hypertension, type II diabetes mellitus, depression, heart disease such as myocardial infarction, cardiac arrhythmia and congestive heart failure, stroke, chronic obstructive pulmonary disease (COPD), osteoarticular disease, cataracts, cancer, thyroid disease, Parkinson’s disease, and dementia [3,4,5]. These are risk factors, some of them with a bidirectional impact, that contribute to dementia development [6,7,8,9,10], frailty, disability, and consequently mortality in old age [11,12]. Chronic kidney disease (CKD) is another very prevalent condition in the elderly population and is associated with an increased risk of mortality, frailty, anaemia, cognitive decline, and cardiovascular events [13,14]. Furthermore, micronutrient deficiencies, especially of vitamin D, B complex, Fe and Zn, are common and have been shown to influence the physical and cognitive health of the elderly [15,16]

Some of these conditions were included in the 2022 report by the Lancet Commission on Dementia [17], which found a model in which 12 modifiable risk factors could prevent 35% of the population’s risk of dementia, some of which can be addressed through primary health care (PHC). These include high blood pressure, diabetes mellitus, obesity, depression, hearing loss, smoking, alcohol consumption (>21 units/week), lower education, physical inactivity, social isolation, head trauma, and air pollution. Level of education, which is in turn an indicator of socioeconomic status, and social interaction have a particular impact on cognitive reserve and will determine poorer physical, mental, and cognitive health [10,18,19]. In short, these are circumstances that affect cognitive resilience [20,21].

Successful ageing, though controversial and criticized in its conceptualization, would therefore not consider the absence of disease, but rather the ability to maintain a good quality of life, adequate cognitive function, active social participation, and adherence to a healthy lifestyle [22]. Lifestyle is a focus of medical and biomedical research on ageing and longevity for several reasons. First, it has been assumed for decades that the heritability of life expectancy accounts for only ≤35% [23]. Second, in regions with a higher frequency of people aged ≥90 years in good physical and mental condition, these individuals live in structured families, avoid smoking, follow diets rich in vegetables, engage in daily physical activity, and participate in social activities [24]. Their nutritional pattern, the so-called Mediterranean diet (MedDiet) given the geographical location, is now used with the objective of preventing cardiovascular disease by reducing hypercholesterolemia, high blood pressure, and obesity [25].

United Nations projections for 2050 for the European region show that the proportion of people aged 65 and over will exceed 30%, and the greatest relative growth will be among people over 80, 40% of whom will live in Europe and North America (109 million). This aligns with data from the Spanish Statistical Office [26,27]. Consequently, healthy ageing, understood as the development and maintenance of functional capacity that enables well-being in old age, constitutes a challenge for healthcare systems [28]. In Spain, the prevalence of dementia among people aged ≥80 years is 25% [29], which means that 75% of this age group could be potentially resilient and should be examined.

For these reasons, in this preliminary study, our objective was to analyse the situation of community-dwelling people aged ≥80 years without a dementia diagnosis in terms of their chronic comorbidities, lifestyle, cognitive status, and autonomy in performing daily tasks. Based on the premise that 80 years of age marks the threshold of cognitive decline, we want to study this age group specifically, as it is normally included within broader age ranges. Understanding its characteristics prepares us for the future, as it will be a population with significant demographic weight. Among them, we chose community-dwelling people without a definitive diagnosis of dementia, based on two circumstances: (i) these people would not be subject to standardized patterns, living with the everyday experiences of most people; (ii) hypothetically, this group should include resilient individuals with conserved cognitive status.

A second goal was to identify and characterize the “healthy ageing” group. However, given their age, the participants might exhibit degrees of dependency or cognitive deficits, complicating the statistical analysis, especially considering the numerous variables studied. Therefore, we employed a multiple equation modelling procedure, latent profile analysis (LPA), to uncover hidden subgroups beyond the group with optimal conditions.

Participants were recruited through PHC for two reasons. First, PHC constitutes an accessible and robust channel for engaging the general population, as individuals are automatically registered and routinely utilize these services, given the characteristics of the Spanish public health system. Second, the results may be of interest for establishing policies for the care of older people, as most risk factors can be addressed from there. Furthermore, we believe that studies need to be carried out in regions such as southern Spain, which have characteristics similar to those of the blue zones of the Mediterranean.

## 2. Materials and Methods

### 2.1. Participants and Procedure

This cross-sectional study was conducted among individuals affiliated with the Las Delicias PC Centre, located in Jerez de la Frontera (southern Spain). The inclusion criteria were as follows: (i) age ≥ 80 years; (ii) not having been diagnosed with dementia; and (iii) not being institutionalized. Cases were randomly recruited between January and June 2025. According to the municipal census, which is mandatory in Spain, in 2022 this city had 212,703 inhabitants, of whom 9373 were of that age (4.4% of the population) [30]. The number of individuals assigned to the Las Delicias centre was 27,579, of whom 1224 were ≥80 years old [31] (the same percentage as that obtained from the Spanish Statistical Office (INE) for the municipality) [30].

**Figure 1:**
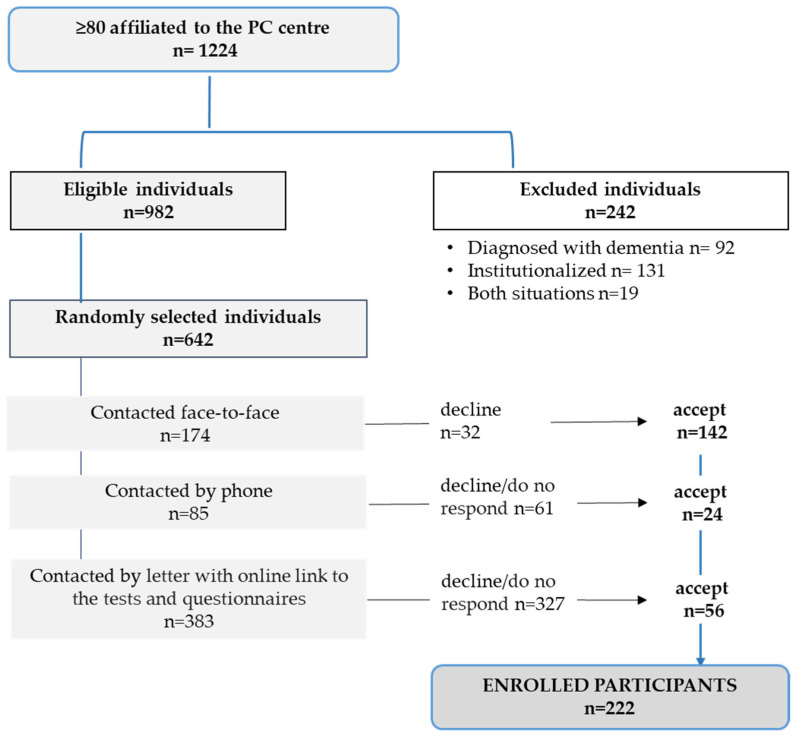
Flowchart indicating the procedure followed to recruit the participants.

Participants were recruited as shown in the flowchart (Figure 1). After being screened using the exclusion criteria, 642 individuals were approached in three ways: in person or by telephone when appointments were scheduled; if not, by regular mail, which contained information for the patient and a link to a Google form with access to the informed consent and the different tests and questionnaires: https://forms.gle/rZgiX3zna2nM4yig8 (accesed on 10 June 2025).

After obtaining informed consent, 222 participants were enrolled, and cognitive, independence, and lifestyle tests were administered. If they required assistance, their accompanying family members or caregivers were informed. Tests and questionnaires were carefully designed by trained members of our team. Information on the chronic comorbidity status and selected blood parameters was then recorded from their electronic medical records. This information had been obtained from previous examinations carried out by their general practitioner (GP).

The sample size obtained allows for 95% confidence intervals for important prevalence parameters, with a maximum margin of error of 5.7%.

### 2.2. Measures and Data Sources

#### 2.2.1. Cognitive Status and Dependence

**Cognitive status** was measured by a revised and Spanish adapted version of the Pfeiffer’s test, a Short Portable Mental Status Questionnaire (SPMSQ), and an easy-to-use tool for primary care, with 10 items (each error is worth 1 point) adjusting the score for school educational level. Thus, the absence of subjective cognitive decline was considered to be <3 errors made <4 errors if less than primary school) [32] (Appendix A.1).

**Autonomy in daily tasks** was assessed by the Spanish translation of the Barthel index (BI), a routine HPC test that contains 10 items that measure functional ability in basic activities of daily living [33]. The final score ranged from 0 to 100; the closer to 100, the greater the independence (Appendix A.2). Individuals are classified into five stages of dependency based on their BI score: total dependency (BI score of ≤20 points); severe dependency (25–60 points); moderate dependency (65–90 points); mild dependency (95 points); and complete independency (BI score of 100).

#### 2.2.2. Lifestyle

Lifestyle was evaluated using questionnaires that included two sets of questions:

(i) The presence of modifiable risk factors, outlined in the Lancet report [17], such as obesity, educational level, physical activity, social support, hearing loss, smoking, and alcohol consumption, was tested as described in Appendix A.3. This intervention provided necessary information under-registered in routine clinical practice, as happens with obesity, despite it being a common chronic condition. To this end, weight and height were requested and subsequently recalculated as body mass index (BMI), expressed in units of kg/m^2^ (values: <18.5, underweight; 18.5–25, normal; >25–30, overweight; ≥30, obese). Education level was divided into less than elementary school, primary/or high school, and university. Physical activity, social support, and hearing loss were assessed following the method proposed by Katayama et al. [34]. Alcohol consumption, also included in MedDiet, was tested separately, according to the risk criteria referred to for dementia [17], with a limit set at 21 units/week (standardized, with 1 unit defined as 10 g of ethyl alcohol).
(ii) Adherence to MedDiet was assessed following the criteria established by Matavelli et al. [35]. It consists of 14 items (each worth 1 point), and adherence is considered to exist if the score is ≥8 (Appendix A.4).

#### 2.2.3. Demographic and Clinical Variables

Demographic variables (sex and age), common chronic conditions, and blood test parameters were collected from electronic medical records. Information about the diagnosis of dementia and institutionalization was extracted from this source. Positive diagnoses (if it listed as such or under treatment) were sought for the following disorders, which are expected to have a high prevalence according to the literature: hypertension (defined as a diastolic/systolic blood pressure reading ≥140 mmHg/≥90 mmHg or treatment for it); diabetes mellitus; cardiovascular diseases (CVD), which would include one or more diagnoses of entities such as myocardial ischemia, cardiac attack, congestive heart failure, atrial fibrillation, valvular disease, peripheral arterial disease, diabetic retinopathy, retinal thrombosis; cerebrovascular diseases (CRVD) such as stroke or transient ischemic attack; chronic kidney disease (CKD); COPD (emphysema, bronchitis); non-cutaneous cancer, skin cancer, osteoarticular diseases (osteoporosis, osteoarthritis); depression, anxiety, insomnia; cataracts; Parkinson’s disease; endocrine system diseases (thyroid and parathyroid conditions); and hyperuricemia. Comorbidity was also calculated as the average number of these conditions. These chronic conditions have been diagnosed by a GP almost or and during the last 12 months.

The results of the latest blood biochemistry analysis were incorporated when available, as some variables are not routinely requested from patients. These included

(i) Blood Parameters associated with cardiovascular risk: fasting glucose, glycosylated haemoglobin (HbA1c), total cholesterol, high-density lipoprotein cholesterol (HDL-c), low-density lipoprotein cholesterol (LDL-c), triglycerides.
(ii) Blood Parameters associated with renal function: creatinine, uric acid.
(iii) Deficiencies: vitamin D, vitamin B12, folic acid, total iron-binding capacity (TIBC). TIBC was the assessment used to diagnose conditions like iron-deficiency anaemia.

### 2.3. Statistical Analysis

Descriptive statistics were applied to analyse the demographic, clinical, and functional information. Categorical variables were expressed as the number and percentage of the observed data, and numeric variables were represented as mean ± standard deviation. Blood levels were recorded as mean ± sd, and deficiencies were also expressed as dichotomised categorical variables (normal vs. deficiency).

The association between categorical variables was assessed using Pearson’s χ^2^ test or, if the conditions were not verified, using Fisher’s exact test. Comparisons between quantitative variables were performed using the Student *t*-test or ANOVA, depending on the number of categories. A two-sided *p*-value of <0.05 was considered statistically significant. Statistical analyses were carried out irrespective of who compiled the database and the GP who provided the anonymized data.

Our objective was to profile the participants and identify potentially resilient individuals so that we could see the distribution of demographic variables, lifestyle, comorbidity, and blood parameters in each of them. But a wide variety of combinations between possible degrees of cognitive impairment and autonomy were expected. To find subgroups, we used LPA. Prior to analysis, four indicators were selected: (i) gender (male/female); (ii) age (in years); (iii) BI, distributed into five categories as described above; (iv) Pfeiffer test results, now represented in five categories based on errors made, with the nuances already expressed: normal (0–2 errors), mild cognitive impairment (3–4 errors); moderate cognitive impairment, pathological (5–7 errors); and significant cognitive impairment, pathological (8–10 errors).

First, we explored the solution with one class to obtain the initial fitting parameters. We then increased the number of profiles until we obtained the best-fitting model, respecting theoretical parsimony [36]. To select the best model, we used several criteria such as the Akaike information criterion (AIC), the Bayesian information criterion (BIC), the sample size-adjusted Bayesian information criterion (SSBIC), and the bootstrap likelihood ratio tests (BLRT). Smaller values for all information criteria (reductions of more than 10 points) were considered improvements in model fit. In addition to these fit statistics, we examined the entropy value. This parameter ranges from 0 to 1: values higher than 0.80 indicate that the groups are well separated [37]. Once the best model was identified, we obtained the probabilities of class membership for each individual, which allowed us to identify a categorical variable within the class by assigning each individual to the class with the highest probability of belonging. This variable enables us to analyse the differences in lifestyle, chronic morbidity, and biochemical variables between groups. We used the Mplus program, version 8.7, to perform LPA [38], and IBM SPSS v29.0 (IBM Corp., Armonk, NY, USA), considering *p*-values < 0.05 to be significant.

## 3. Results

Participant characteristics are presented in Table 1, Table 2 and Table 3 according to age and gender distribution, in anticipation of the potential influence of demographic variables on the other parameters analysed. The mean age of the sample was 84.58 years ± 3.72, with a median of 84 years (range: 80–96 years) and a 95% CI of 84.08–85.07. Women accounted for 56.3% (n = 125). Age was similar between the sexes: 84.50 ± 3.81 years (95% CI: 83.83–85.18) for women and 84.67 ± 3.6 years (95% CI: 83.93–85.40) for men.

Cognitive status was preserved in 81.1% (n = 180) of the individuals, who were significantly younger than those with cognitive impairment (84.31 ± 3.64 years vs. 85.71 ± 3.92 years; *p* = 0.024). Among men, 91.8% belonged to the former group (n = 89), compared with 72.8% of women (n = 91) (*p* = 0.000). Regarding autonomy in daily life, 42.7% (n = 95) of the sample was fully independent (BI = 100), being significantly male: 58.8% of men (n = 57) vs. 30.4% of women (n = 38). Women integrated the sub-groups with increasing levels of dependency to a greater extent than men (*p =* 0.000) (Table 1). Age also had a significant influence: the independent group was younger (mean 83.98 ± 3.31 years) than the totally dependent group (86.33 ± 5.13 years; *p* = 0.023).

Concerning lifestyle (Table 1), it is noteworthy that the participants reported that 97.7% (n = 217) had social support, 94.6% (n = 210) consumed alcohol at levels considered low-risk for dementia, 85.1% (n = 189) followed the MedDiet, 74.8% (n = 166) engaged in physical activity, and 72.5% had never smoked. However, 54.5% (n = 121) had not completed primary education. Gender differences indicated that men had higher academic attainment, were engaged in physical activity, and were more likely to consume alcohol and smoke. Thus, 57.7% of men (n = 56) had completed at least primary studies vs. 36% of women (n = 45) (*p* = 0.005); 90.7% of men (n = 88) were physically active vs. 62.4% of women (n = 78) (*p* < 0.001); 96.8% of women (n = 121) had never smoked vs. 41.2% of men (n = 40) (*p* = 0.000); and 98.4% of women (n = 123) showed low-risk alcohol consumption vs. 89.7% of men (n = 87) (*p* = 0.004). Age was significantly associated with hearing loss, which affected older individuals (85.55 ± 3.88 years) compared to those who retained it (83.94 ± 3.48 years) (*p* = 0.002), and related to a higher frequency of physical activity among younger individuals (trend toward significance, *p* ≈ 0.05).

In relation to chronic comorbidity (Table 2), the most frequent conditions were hypertension (86.5%, n = 192), cataracts (74.3%, n = 165), osteoarticular diseases (68.5%, n = 152), and CVD (49.5%, n = 110). About a third of the participants suffered from diabetes (36.0%, n = 80) or obesity (32.9 n = 73) (Table 1). Parkinson’s disease was rare, affecting only 1.4% of the sample. Prevalence varied significantly according to gender for three types of morbidities. COPD, though infrequent (4.1% of sampled individuals), affected only men (9.3%, n = 9) (*p* = 0.001) who were significantly older (87 ± 4.55 years) (*p* = 0.046). Osteoarticular diseases were diagnosed in 84.8% of women (n = 106) vs. 47.4% (n = 46) of men (*p* = 0.000). Women were more affected psychologically: 32.8% (n = 41) had depression vs. 10.3% of men (n = 10) (*p* = 0.000); 19.2% (n = 24) suffered from insomnia vs. 5.2% of men (n = 5) (*p* = 0.002); and 28.8% (n = 36) suffered from anxiety vs. 12.4% (n = 12) of men (*p* = 0.003). On the other hand, 82.9% had ≥4 chronic conditions, with an average number of 6.01 ± 2.43 per individual, being significantly lower in men (5.58 ± 2.44) (*p* = 0.019).

Next, haematological parameters from the most recent blood analyses were compared (Table 3). Blood glucose-related parameters were altered (mean values of fasting glucose 103.88 mg/dL, HbA1c 6.32% in the prediabetes range). The rest of the blood indicators were within the reference range. Lipid values were out of cardiovascular risk range, with notable high levels in all cholesterol-related parameters in women (*p* ≤ 0.001): mean total cholesterol 193.3 mg/dL in women vs. 168.4 in men; HDL-c 56.45 mg/dL vs. 45.28; LDL-c 115.5 mg/dL vs. 100.9. Average levels of creatinine and uric acid were significantly higher in men, as is expected (*p* ≤ 0.001), but within the normal range.

Those suspected of hypovitaminosis or Fe deficiency had an analytical request. Average levels were generally within the normal range for the sampled individuals, although men had lower levels of vitamin B and TIBC (*p* = 0.008 and *p* = 0.01). In fact, deficiencies were rare and did not affect genders differently: B-complex deficiency affected 7% of those studied (n = 10) (with regard to vitamin B12) and 3.6% (n = 5) (with regard to folic acid); 3.8% (n = 6) exhibited high TIBC levels, although these individuals were significantly older (*p* = 0.022). However, 47.9% (n = 34) had Vitamin D deficiency.

Regarding age, Pearson’s correlation showed that triglyceridemia (*p* = 0.006) and haemoglobin glycosylation (close to significance, *p* = 0.056) decreased with age.

### Sample Profiling

Once the LPA is applied, the fit criteria for each model are shown in Appendix B, Table A1. We observed that BIC indicated three profiles, while SSBIC increased this to four profiles, with negligible differences with respect to the five-profile model (a decrease of 0.826). We observed that the AIC and SSBIC decreased when we increased the number of profiles from one to four. By contrast, the pBLRT was significant for the four-profile (*p* = 0.000) and five-profile (*p* = 0.048) models, but not significant (*p* = 0.400) for the six-profile model. Finally, the highest entropy value (0.844) led us to choose the four-profile model.

Then, after LPA analysis (Appendix B Table A2), we observed that profiles 3 and 4 showed no probability of independence, while profiles 1 and 2 had a probability of being cognitively healthy greater than 87% (92% in profile 2). Subsequently, in the search for autonomous individuals without cognitive impairment, we extracted from Profiles 1 and 2 those who met these criteria, creating a ‘healthy ageing’ subgroup (Profile 0; 38.29%, n = 85; 60% men; mean age 84.01 years). This subgroup was compared with the four profiles obtained from the LPA after extraction, hereafter referred to as Profiles A–D. We defined the membership profile as follows: Profile 0—healthy ageing group; Profile A—mild to moderate dependency, majority cognitively normal; Profile B—mild to severe dependency, majority cognitively normal; Profile C—moderate to severe dependency, one-third with mild cognitive impairment; Profile D—moderate to severe dependency, with half moderate and half severe dependency, and one-third with moderate cognitive impairment (Table 4).

A first notable finding is the rise in the loss of daily autonomy with age, as indicated by the increasing probability of severe dependency (BI score 25–60): 12.7% in profile A (mean age 82.23 years); 26.7% in profile C (86.9 years); 29.4% in profile B (88.6 years); and 45.5% in profile D (93.2 years). Secondly, clearer differences can be seen in the association between sex, age, and functionality in the extreme profiles. The “heathy ageing” group was younger (average 84.01 years), predominantly men (60%), and 100% independent, with no cognitive impairment. In contrast, members of profiles C and D were older (conditional probability mean age 86.9 and 93.2 years, respectively) and mostly comprised women (100% and 81.8%, respectively). Also, a correlation was observed between poorer cognitive state and a greater levels of dependence: in profile C, there was a 93% probability of suffering from moderate or severe dependence (already 26% severe), along with a 33% probability of suffering from mild cognitive impairment; members of profile D, who were worse off, had a 91% probability of suffering from moderate or severe dependence (45.5% severe) and a 36% probability of suffering from moderate cognitive impairment (already pathological).

Then, we analysed the distribution of inter-profile variables related to lifestyle and comorbidities. In terms of lifestyle, we observed significance in the decrease of physical activity as the likelihood of severity of dependency increased (*p* = 0.000). Furthermore, a higher frequency of non-smokers was found in the worst profiles (possibly explainable by the fact that these profiles predominantly comprised women) (*p* = 0.001). Although the comparison between the groups gives *p* values of 0.109, it is observed that the prevalence of hearing loss increases with degree of dependency (Table 5).

Concerning the comorbidities, regardless of the visible divergence between profile 0, which was favourable, and profile D, we observed a significantly different distribution of prevalence in CKD, COPD, osteoarticular diseases, anxiety, and, close to significance, depression.

It is worth noting that osteoarticular diseases were less frequent in subgroups with a high percentage of cognitively normal individuals (profile 0, 57.6% and profile B, 58.8%) compared to other profiles: profile A, 75.9%; profile C, 86.6%; and profile D, 63.6% (*p* = 0.017). Also, profiles 0 and B were profiles with male predominance. Anxiety followed a similar pattern (*p* = 0.021), as did depression (*p* = 0.067). Due to the small sample size (n = 9), the distribution of the prevalence of COPD makes interpretation difficult, but it is true that the majority (n = 7) are in non-“healthy” profiles (*p* = 0.05). Finally, we observe that the number of comorbidities increases as the likelihood of having a worse result on the Pfeiffer test increases, with the greatest difference between profile 0 (mean 5.22 ± 2.34) and profile D (mean 7.18 ± 2.48) (*p* = 0.002) (Table 6).

Blood biochemical parameters were similar across all profiles except for sex-corrected creatinine, which was higher in the older profiles: profile B, 1.44 ± 1.19 (probable mean age 88 years), and profile D, 1.13 ± 0.53 (96 years) (*p* = 0.008) (Table 7).

## 4. Discussion

The aim of this study was to determine the condition of dementia-free, community-dwelling individuals aged 80 years and older and to identify those susceptible of “healthy ageing,” defined as people who have retained their cognitive capacity and full autonomy in daily tasks. From the descriptive analysis, we found that 81.1% of the participants were cognitively healthy, 42.7% were completely independent, women showed significantly worse conditions, and age was associated with deterioration in these two parameters. 

Given all possible combinations of the Pfeiffer results, BI score ranges, sex, and age, we resorted to a multiple equation calculation strategy—LPA—which yielded a four-profile model. Subsequently, we extracted a fifth profile, or profile 0, from individuals who met the ‘healthy ageing’ criteria, representing 38.3% of the sample, of whom 60% were men. We present a summary of the statistical significance of variable distribution in Appendix B, Table A3.

The first step is to contextualize this finding. A study of 2305 community-dwelling adults (mean age 84.6 ± 7.0 years) showed that 47.7% of them exhibited a non-frail phenotype (disability and comorbidity included in the index), and this group predominantly comprised men (55.6%), in line with our data [12].

Another study conducted in a similar Spanish population [39] obtained worse results, with more than 60% of participants reporting significant functional dependence for ADLs and IADLs (BI score ≤90 in 57.3% of our sample) and 51.9% experiencing cognitive impairment (18.9% in our sample). The social vulnerability of these patients (51.5% showed such a condition and 17.0% lived alone) could be a factor that explains this better than age (they were older; mean age, 88 years), while it is noteworthy that our participants felt socially supported in 97% of cases, possibly in addition to generally following a “healthy” lifestyle. Thus, another study found in its sample a subgroup defined as “consistent engagement in healthy behaviors” that shared characteristics with ours: physically active individuals (82% vs. 74.8% in our sample), never smokers (70% vs. 72.5%), non-drinkers (81% vs. 94.6%), and eating fresh vegetables daily (82% vs. 85% adhering to the MedDiet) [40].

That is, our study shows a high percentage of people with good cognitive and functional status, consistent with the signs of a healthy lifestyle in the group [41]. This would not be unusual, as older people tend to have a good lifestyle [42]. Besides, that may be expected in populations in the Mediterranean area.

With regard to chronic morbidities, we cannot affirm that our participants are in a better or worse situation than others of the same age. Hypertension tops the list, followed by osteoarticular diseases, heart diseases, cataracts, and diabetes, which, as in our work, tend to be the most frequent [11]. Comparisons across studies are limited by differences in nosological classifications and age ranges. For instance, obesity and hypertension showed higher prevalence in our sample (33% and 86%, respectively) compared with another parallel study (in which it ranged between 20–25% and 60–70%, respectively), whereas the occurrence of CRVD was lower (10% vs. ~25%) [43]. These studies agree with us in reporting significant gender differences: women exhibited a higher prevalence of osteoarticular diseases and psychological symptoms, while men presented higher rates of COPD and CVD [11]. Moreover, we observed that the average number of comorbidities in men (~5) was similar to that observed in other studies, while in women it was higher (6) (*p* = 0.021) [43]. As for blood biochemistry related to carbohydrates and lipids, the values fell within the expected ranges for advanced age [44,45].

Analysing the statistical significance of the inter-profile distribution of the variables, we observed that the percentage of physically active individuals decreased as the severity of dependency increased (*p* = 0.000). Numerous studies point to advanced age, female sex, and lower levels of physical activity as predictors, among other factors, of the development of dependency [46], as well as the interaction between disability, preserved cognition, and lifestyle [47], particularly linking physical activity to cognitive preservation [48]. The profiles that most closely align with this finding are C and D, showing a higher likelihood of the joint presence of greater disability (BI score <60) and poor results in the Pfeiffer test. These profiles mostly comprise women (100% in profile C and 82% in D), representing 18% of the sample. However, we cannot assert which is the cause and which is the effect.

Moreover, cognitive impairment appears to be associated with sex rather than age. Perhaps the analysis of the next group of comorbidities may shed light. In this regard, the distribution of osteoarticular diseases (*p* = 0.017) and psychological conditions—specifically anxiety (*p* = 0.021) and depression (approaching significance, *p* = 0.067)—differed significantly across profiles. Previous research has suggested that the association between cognitive impairment and osteoarticular disease may be mediated by depression and anxiety [49]. Additionally, treatment patterns for these conditions are frequently reported to be more common among women [50]. In our study, these pathologies were more prevalent in predominantly female profiles, which exhibited a higher likelihood of cognitive impairment (profile A: 77% probability that the individuals were cognitively healthy; 46% in profiles C and D) (Table 6). The literature strongly links both osteoarticular and psychopathological factors to disability and lower quality of life [11], but in our study, this is not the case. Profile A, consisting of the youngest members (probable mean age of 82 years) with a high prevalence of osteoporosis (76%), depression (31.6%), and anxiety (21,5%), shows the lowest levels of disability among profiles A–D (a 76% probability of mild or moderate disability).

Concerning other chronic conditions with statistical significance, profile D had a fourfold higher probability of suffering from CKD (36.3%) compared to profile 0 (9.4%) (*p* = 0.046). Average creatinine levels were also significantly high, following the same prevalence pattern (*p* = 0.008). CKD is currently considered a common chronic disease, with an increasing prevalence with age, and it is associated not only with cardiovascular risk factors but also with the risk of physical and cognitive dysfunction [13]. However, the prevalence of CKD observed in our sample as a whole, as well as in the remaining profiles, was lower than that found in the literature (29–37.5% in individuals over 65–70 years of age), being comparable only to profile D (although individuals in this profile were 93 years old) [14,51]. Only 2 of the 9 individuals affected by COPD were in profile 0 (*p* = 0.050). It should also be noted that although there were no significant differences between the groups (*p* = 0.205), profile D exhibited CVD in 81% of cases, which is almost double that of the other profiles. And as could be expected, a significant difference in the number of chronic comorbidities was observed (*p* = 0.002), with the greatest difference found between the extreme profiles (0: 5.22 vs. D: 7.18).

It is worth noting that the risk factors associated with the onset of dementia and disability—such as alcohol consumption, diabetes, obesity, hypertension, or low educational attainment ([11,17,34])—did not differ significantly across profiles. Conversely, individuals with greater dependency and cognitive impairment (profiles C and D) were highly unlikely to have ever smoked (*p* = 0.001). This was possibly linked to the fact that most of them were women, who generally do not smoke. Finally, remarkable differences, with or without statistical significance, were observed between profiles 0 and D: profile 0 consisted of 100% independent, 100% cognitively healthy individuals, aged about 84 years old, with 60% being men; by contrast, 90% of the individuals in profile D (5% of the sample) had moderate or severe dependence (split evenly), 36% had pathological cognitive impairment, 82% were women, and the average age was 93 years.

Regarding the source used in this study for recruitment and for clinical data, we believe it is essential to conduct the research on-site, rather than using data from a general information system database [52]. Primary healthcare services in Spain are particularly valuable due to the personalized care they provide to patients and the availability of accurate information. Nevertheless, we know that this approach has limitations. It is not possible to establish causal relationships between functional and cognitive status and the selected variables, because we do not know how long they have suffered from their conditions or how long they have been maintaining a healthy lifestyle. On the other hand, people in excellent health may not have been interested in participating, and those in very poor health may not have been able to do so. Being older, they may not have had a precise memory of what was being asked, although they were helped by their relatives during the interviews.

Furthermore, the small sample size led us to minimize subdivisions as much as possible (e.g., age ranges, disease types among the main nosological entities) for statistical analysis. However, it is equally true that obtaining large samples for these age groups also poses difficulties. To address these limitations, we plan to recruit new participants and perform additional measurements to more accurately assess each individual’s condition: (i) expanding biochemical and haematological tests to determine blood cells, organ function biomarkers, and ions; (ii) analysing molecular ageing biomarkers, such as relative telomere length, FOXO3A polymorphisms, and APOE alleles ε2, ε3, and ε4—which also serve as indicators of dementia predisposition; and (iii) evaluating preclinical markers of dementia [44,53].

## 5. Conclusions

In this preliminary study, we analysed a community-dwelling, dementia-free population of people aged 80 living in an Andalusian city, and therefore in a Mediterranean context. The group studied as a whole seemed to practice a healthy lifestyle as self-reported, despite the fact that their health status was not better than in other studies. After performing an LPA, we found that 38.29% of the participants met the requirements of healthy ageing (without cognitive deterioration and total independence) and were predominantly male. By contrast, individuals with a higher probability of more severe dependence had a high probability of cognitive impairment and were likely female, representing 18.5% of the sample. The probability of suffering from severe dependence was associated with increasing age among the profile members, although cognitive status preservation was associated with being male. Physical activity, smoking, osteoporosis, anxiety, COPD, CKD, creatinine blood levels, and number of comorbidities exhibited statistical differences in the inter-profiles. Finally, men were in a better position than women in important aspects of lifestyle, had fewer chronic comorbidities, and were more likely to be resilient to ageing. These findings pose several challenges for primary care. Special attention should be paid to CKD due to its prevalence and its potentially debilitating effect. Secondly, it is necessary to routinely assess the cognitive status of older patients; some people escape diagnosis of dementia if this is not done. Thirdly, women live the longest, but their health and functionality are severely impaired. Healthcare services for elderly women should prioritize managing joint pain and mental health and promoting an active lifestyle.

## Figures and Tables

**Table 1:** Cognitive status, autonomy, and lifestyle of the participants by gender and age.

	Total n = 222	Men n = 97	Women n = 125		Age	
	n (%)			*p*	Mean ± sd	*p*
**Cognitive status** n (%)				**0.000**		**0.024**
Normal	180 (81.1)	89 (91.8)	91 (72.8)		84.31 ± 3.64	
Cognitive impairment	42 (18.9)	8 (8.2)	34 (27.2)		85.71 ± 3.92	
**Autonomy**				**0.000**		**0.023**
Independence	95 (42.7)	57 (58.8)	38 (30.4)		83.98 ± 3.31	
Mild dependence	35 (15.8)	16 (16.5)	19 (15.2)		83.77 ± 3.32	
Moderate dependence	61 (27.5)	17 (17.5)	44 (35.2)		85.16 ± 3.79	
Severe dependence	28 (12.6)	7 (7.2)	21 (16.8)		86.14 ± 4.66	
Total dependence	3 (1.4)	0 (0)	3 (2.4)		86.33 ± 5.13	
**Education** n (%)				**0.005**		0.820
Less than primary school	121 (54.5)	41 (42.3)	80 (64)		84.64 ± 3.69	
Primary or high school	86 (38.7)	47 (48.4)	39 (31.2)		84.58 ± 3.83	
University	15 (6.8)	9 (9.3)	6 (4.8)		84 ± 3.50	
**Physical activity**				**0.000**		0.052
No	56 (25.2)	9 (9.3)	47 (37.6)		85.41 ± 4.30	
Yes	166 (74.8)	88 (90.7)	78 (62.4)		84.29 ± 3.47	
**Social support**				0.280		0.388
Yes	217 (97.7)	96 (99)	121 (96.8)		84.54 ± 3.71	
No	5 (2.3)	1 (1)	4 (3.2)		86 ± 4.24	
**Hearing loss**				0.167		**0.002**
No	135 (60.8)	54 (55.7)	81 (64.8)		83.94 ± 3.48	
Yes	87 (39.2)	43 (44.3)	44 (35.2)		85.55 ± 3.88	
**Smoking**				**0.000**		0.460
No	161 (72.5)	40 (41.2)	121 (96.8)		84.72 ± 3.77	
Ex-smokers	55 (24.8)	52 (53.6)	3 (2.4)		84.32 ± 3.58	
Daily smokers	6 (2.7)	5 (5.2)	1 (0.8)		83 ± 3.63	
**Alcohol consumption**				**0.004**		0.869
No ^§^	210 (94.6)	87 (89.7)	123 (98.4)		84.56 ± 3.75	
Yes	12 (5.4)	10 (10.3)	2 (1.6)		84.75 ± 3.30	
**MedDiet adherence**				0.173		0.723
No	33 (14.9)	18 (18.6)	15 (12)		84.36 ± 3.40	
Yes	189 (85.1)	79 (81.4)	110 (88)		84.61 ± 3.78	

^§^ Alcohol consumption not associated with dementia risk.

**Table 2:** Distribution of common chronic health conditions by gender and age.

	Total n = 222	Men n = 97	Women n = 125		Age	
		n (%)		*p*	Mean ± sd	*p*
**BMI**				0.319		0.724
Normal	56 (25.2)	20 (20.6)	36 (28.8)		84.80 ± 3.45	
Overweight	93 (41.9)	45 (46.4)	48 (38.4)		84.34 ± 3.87	
Obesity	73 (32.9)	32 (33)	41 (32.8)		84.69 ± 3.76	
**Hypertension**				0.661		0.183
No	30 (13.5)	12 (12.4)	18 (14.4)		83.73 ± 3.22	
Yes	192 (86.5)	85 (87.6)	107 (85.6)		84.70 ± 3.78	
**Diabetes**				0.990		0.623
No	142 (64.0)	62 (63.9)	80 (64)		84.66 ± 3.76	
Yes	80 (36.0)	35 (36.1)	45 (36)		84.41 ± 3.66	
**CVD**				0.060		0.527
No	112 (50.5)	42 (43.3)	70 (56)		84.41 ± 3.41	
Yes	110 (49.5)	55 (56.7)	55 (44)		84.73 ± 4.02	
**CRVD**				0.982		0.452
No	199 (89.6)	87 (89.7)	112 (89.6)		84.51 ± 3.71	
Yes	23 (10.4)	10 (10.3)	13 (10.4)		85.13 ± 3.84	
**CKD**				0.733		0.356
No	183 (82.4)	79 (81.4)	104 (83.2)		84.46 ± 3.57	
Yes	39 (17.6)	18 (18.6)	21 (16.8)		85.07 ± 4.34	
**COPD**				**0.001**		**0.046**
No	213 (95.9)	88 (90.7)	125 (100)		84.47 ± 3.66	
Yes	9 (4.1)	9 (9.3)	0 (0)		87 ± 4.55	
**Cancer**				0.145		0.365
No	150 (67.6)	61 (62.9)	89 (71.2)		84.51 ± 3.59	
Non-skin cancer	44 (19.8)	19 (19.6)	25 (20)		84.22 ± 3.98	
Skin cancer	28 (12.6)	17 (17.5)	11 (8.8)		85.46 ± 3.99	
**Osteoarticular diseases**				**0.000**		0.738
No	70 (31.5)	51 (52.6)	19 (15.2)		84.7 ± 4.05	
Yes	152 (68.5)	46 (47.4)	106 (84.8)		84.51 ± 3.56	
**Depression**				**0.000**		0.720
No	171 (77.0)	87 (89.7)	84 (67.2)		84.62 ± 3.73	
Yes	51 (23.0)	10 (10.3)	41 (32.8)		84.41 ± 3.69	
**Insomnia**				**0.002**		0.318
No	193 (86.9)	92 (94.8)	101 (80.8)		84.67 ± 3.77	
Yes	29 (13.1)	5 (5.2)	24 (19.2)		83.93 ± 3.32	
**Anxiety**				**0.003**		0.850
No	174 (78.4)	85 (87.6)	89 (71.2)		84.55 ± 3.80	
Yes	48 (21.6)	12 (12.4)	36 (28.8)		84.66 ± 3.42	
**Cataracts**				0.779		**0.035**
No	57 (25.7)	24 (24.7)	33 (26.4)		83.68 ± 3.97	
Yes	165 (74.3)	73 (75.3)	92 (73.6)		84.88 ± 3.59	
**Endocrine disease**				0.086		0.750
No	203 (91.4)	92 (94.8)	111 (88.8)		84.60 ± 3.74	
Yes	19 (8.6)	5 (5.2)	14 (11.2)		84.32 ± 3.64	
**Hypothyroidism**				0.081		0.957
No	205 (92.3)	93 (95.9)	112 (89.6)		84.58 ± 3.72	
Yes	17 (7.7)	4 (4.1)	13 (10.4)		84.52 ± 3.76	
**Hyperuricemia**				0.059		0.287
No	166 (74.8)	67 (69.1)	99 (79.2)		84.42 ± 3.71	
Yes	56 (25.2)	30 (30.9)	26 (20.8)		85.04 ± 3.76	
**Parkinson’s disease**				1.000		0.611
No	219 (98.6)	96 (99)	123 (98.4)		84.56 ± 3.71	
Yes	3 (1.4)	1 (1)	2 (1.6)		85.67 ± 5.51	
**Comorbidity**				**0.019**	R *	0.052
Average number	6.01 ± 2.43	5.58 ± 2.44	6.34 ± 2.38		0.062	0.358

* R: Pearson correlation.

**Table 3:** Blood biochemistry results of participants by gender and age.

	Total	Men	Women		Age	
	mean ± sd ^§^			*p*	R */mean ± sd	*p*
**Cardiovascular risk factors**						
Fasting glucose (n = 205)	103.88± 27.15	103.3 ± 25.8	104.2 ± 28.1	0.825	−0.046 *	0.511
HbA1c (n = 155)	6.32 ± 1.04	6.326 ± 0.91	6.320 ± 1.12	0.970	−0.154 *	**0.056**
Total cholesterol (n = 199)	182.73 ± 37.27	168.4 ± 34.0	193.3 ± 36.0	**0.000**	−0.009 *	0.898
HDL-c (n = 197)	51.63 ± 14.92	45.28 ± 10.5	56.45 ± 15.9	**0.000**	0.082 *	0.254
LDL-c (n = 197)	109.23 ± 31.64	100.9 ± 29.9	115.5 ± 31.5	**0.001**	0.006 *	0.929
Triglycerides (n = 199)	120.61 ± 56.57	123.6 ± 65.4	118.3 ± 49.1	0.518	−0.196 *	**0.006**
**Kidney function**						
Creatinine (n = 203)	1.05 ± 0.47	1.177 ± 0.57	0.959 ± 0.35	**0.001**	0.071 *	0.316
Uric acid (n = 190)	5.76 ± 1.7	6.430 ± 1.64	5.264 ± 1.57	**0.000**	0.081 *	0.267
**Deficiencies** ^†^						
**Vitamin B12** (n = 143)	357.36 ± 148.29	319.33 ± 119.24	382.5 ± 160.49	**0.008**	−0.049 *	0.558
deficiency (n; %)				0.155		0.384
No	133 (93)	51 (89.5)	82 (95.3)		84.59 ± 3.86	
Yes	10 (7.0)	10 (10.5)	10 (4.7)		85.70 ± 4.24	
**Folic acid** (n = 137)	7.44 ± 3.72	6.99 ± 3.51	7.68 ± 3.52	0.254	0.109 *	0.204
deficiency (n; %)				0.670		0.386
No	132 (96.4)	54 (96.4)	78 (96.3)		84.76 ± 3.97	
Yes	5 (3.6)	2 (3.6)	3 (3.7)		83.20 ± 1.92	
**Vitamin D** (n = 71)	24.49 ± 15.76	26.61 ± 16.13	23.47 ± 15.64	0.435	−0.009 *	0.939
deficiency (n; %)				0.126		0.079
No	37 (52.1)	15 (65.2)	22 (45.8)		83.24 ± 3.49	
Yes	34 (47.9)	8 (34.8)	26 (54.2)		84.82 ± 3.97	
**Fe (TIBC)** (n = 158)	331.22 ± 59.17	317.09 ± 56.88	341.62 ± 58.96	**0.010**	−0.034 *	0.673
altered availability (n; %)				0.494		**0.022**
No	152 (96.2)	65 (97.0)	87 (95.6)		84.45 ± 3.65	
Yes	6 (3.8)	2 (3.0)	4 (4.4)		88.00 ± 4.73	

* R = Pearson correlation. ^§^ Normal range and units of measurement: **Fasting glucose**: 74–100 mg/dL; **HbA1c** 4.7–5.6%, normal; 5.7–6.4%, pre-diabetes; ≥6.5% diabetes. **Total cholesterol** 1–200 mg/dL; **HDL-c** 50–80 mg/dL, normal; <40 ♂, cardiovascular risk; <45 ♀, cardiovascular risk; **LDL-c** 100–130 mg/dL, normal; >130, cardiovascular risk; **triglycerides** 4–150 mg/dL. **Vitamin D** 20–89 ng/mL; **Vitamin B12** 187–883 pg/mL; **Folic acid** 3.1–20 ng/mL; **TIBC** (total iron-binding capacity) 250–450 μg/dL (a high level suggests low iron availability). **Creatinine** 0.72–1.26 mg/dL ♂; 0.57–1.1 mg/dL ♀. **Uric acid** 3.5–7.2 mg/dL ♂; 2.6–6 mg/dL ♀. ^†^ Percentages calculated among tested patients only.

**Table 4:** Description of profiles after extracting the cognitively healthy group from the four-profile model.

	Profile 0 ^§^	Profile A ^¥^	Profile B ^¥^	Profile C	Profile D
n	85	79	17	30	11
prevalence (%)	38.29%	35.59%	7.66%	13.51%	4.96%
Men (%)	60.0%	38%	82.4%	0%	18.2%
Mean age (years)	84.01	82.23	88.59	86.93	93.18
	real values	conditional probability values			
BI score range					
0	**1**	0.101	0.118	0	0
1	0	**0.342**	**0.412**	0.033	0
2	0	**0.418**	**0.176**	**0.667**	**0.455**
3	0	0.127	**0.294**	**0.267**	**0.455**
4	0	0.013	0	0.033	0.091
Categorized Pfeiffer results					
0	1	**0.772**	**0.824**	**0.467**	**0.455**
1	0	0.101	0.176	**0.333**	0.182
2	0	0.127	0	0.100	**0.364**
3	0	0	0	0.100	0
					
**Profile definition**	**Healthy ageing**	**Mild moderate-dependency** **Majority** **cognitively healthy**	**Mild-severe dependency** **Majority** **cognitively healthy**	**Moderate-severe dependency** **(26% severe)** **33% mild cognitive** **impairment**	**Moderate- severe dependency** **(half and half)** **36% moderate cognitive impairment**
dependency	100% independency	75% mild or moderate	59% mild or moderate 29% severe	93% moderate or severe (26.7% severe)	91% moderate or severe (45.5% moderate 45.5% severe)
cognition	100% normal	77% normal	82% normal	47% normal 33% mild	46% normal, 36% moderate

^§^ Profile 0 = Individuals independent and cognitively normal (BI score range = 0; Pfeiffer category = 0); ^¥^ Profile resulting from extraction of individuals independent and with normal cognition.

**Table 5:** Lifestyle characteristics across profiles.

	TOTAL	Profile 0	Profile A	Profile B	Profile C	Profile D	
n	222	N = 85	N = 79	N = 17	N = 30	N = 11	
Prevalence (%)	100%	38.29%	35.59%	7.66%	13.51%	4.96%	
Male (%)	43.7%	60.0%	38%	82.4%	0%	18.2%	
Mean age (years)	84.58	84.01	82.23	88.59	86.93	93.18	
			n (%)				*p*
**Education**							0.096
Less than primary school	121 (54.5)	46 (54.1)	40 (50.6)	6 (35.2)	24 (80)	5 (45.4)	
Primary or high school	86 (38.7)	33 (38.8)	32 (40.5)	10 (58.8)	6 (20)	5 (45.4)	
University	15 (6.8)	6 (7)	7 (8.8)	1 (5.8)	0 (0)	1 (9)	
**Hearing loss**	87 (39.2)	30 (35.2)	26 (32.9)	8 (47)	16 (53.3)	7 (63.6)	0.109
**Physically active**	166 (74.8)	76 (89.4)	58 (73.4)	12 (70.5)	16 (53.3)	4 (36.3)	**0.000**
**Socially supported**	217 (97.7)	83 (97.6)	78 (98.7)	16 (94.1)	30 (100)	10 (90.9)	0.276
**Never smoking**	161 (72.5)	57 (67)	55 (69.6)	9 (52.9)	30 (100)	10 (90.9)	**0.001**
**Alcohol consumption ^§^**	210 (94.6)	77 (90.5)	76 (96.2)	16 (94.1)	30 (100)	11 (100)	0.304
**Adherence to MedDiet**	189 (85.1)	74 (87)	64 (81)	15 (88.2)	27 (90)	9 (81.8)	0.740

^§^ Alcohol consumption not associated with risk.

**Table 6:** Distribution of common chronic health conditions across profiles.

	TOTAL	Profile 0	Profile A	Profile B	Profile C	Profile D	
n	222	85	79	17	30	11	
Prevalence (%)	100%	38.29%	35.59%	7.66%	13.51%	4.96%	
Male (%)	43.7%	60.0%	38%	82.4%	0%	18.2%	
Mean age (years)	84.58	84.01	82.23	88.59	86.93	93.18	
			n (%)				*p*
**BMI**							
Normal	56 (25.2%)	29 (34.1)	14 (17.7)	4 (23.5)	7 (23.3)	2 (18.1)	0.352
Overweight	93 (41.9%)	34 (40)	37 (46.8)	8 (47)	10 (33.3)	4 (36.3)	
Obesity	73 (32.9%)	22 (25.8)	28 (35.4)	5 (29.4)	13 (43.3)	5 (45.4)	
**Hypertension**	192 (86.5%)	73 (85.8)	67 (84.8)	16 (94.1)	25 (83.3)	11 (100)	0.672
**Diabetes**	80 (36.0%)	24 (28.2)	36 (45.5)	8 (47)	8 (26.6)	4 (36.3)	0.116
**CVD**	110 (49.5%)	44 (51.7)	37 (46.8)	7 (41.1)	13 (43.3)	9 (81.8)	0.205
**CRVD**	23 (10.4%)	6 (7)	10 (12.6)	3 (17.6)	3 (10)	1 (9)	0.578
**CKD**	39 (17.6%)	8 (9.4)	19 (24)	3 (17.6)	5 (16.6)	4 (36.3)	**0.046**
**COPD**	9 (4.1%)	2 (2.3)	3 (3.7)	3 (17.6)	0 (0)	1 (9)	**0.050**
**Cancer**	72 (32.4%)	23 (27)	29 (36.7)	5 (29.4)	10 (33.3)	5 (45.4)	0.607
**Osteoarticular**	152 (68.5%)	49 (57.6)	60 (75.9)	10 (58.8)	26 (86.6)	7 (63.6)	**0.017**
**Depression**	51 (32.4%)	15 (17.6)	25 (31.6)	1 (5.8)	6 (20)	4 (36.3)	0.067
**Insomnia**	29 (13.1%)	10 (11.7)	12 (15.1)	0 (0)	5 (16.6)	2 (18.1)	0.398
**Anxiety**	48 (21.6%)	13 (15.2)	17 (21.5)	2 (11.7)	13 (43.3)	3 (27.2)	**0.021**
**Cataracts**	165 (74.3%)	61 (71.7)	55 (69.6)	15 (88.2)	26 (86.6)	8 (72.7)	0.254
**Endocrine** **diseases**	19 (8.6)	6 (7.1)	7 (8.9)	2 (11.8)	2 (6.7)	2 (18.2)	0.634
Hypothyroidism	17 (7.7)	5 (5.9)	6 (7.6)	2 (11.8)	2 (6.7)	2 (18.2)	0.491
**Hyperuricemia**	56 (25.2)	18 (21.2)	21 (26.6)	6 (35.3)	8 (26.7)	3 (27.3)	0.775
**Parkinson**	3 (1.4)	1 (1.2)	1 (1.3)	0 (0.0)	1 (3.3)	0 (0.0)	0.698
	Average number ± sd						
**Comorbidity**	6.01 ± 2.43	5.22 ± 2.34	6.59 ± 2.38	6.00 ± 2.29	6.27 ± 2.36	7.18 ± 2.48	**0.002**

**Table 7:** Comparison of blood biochemistry parameter levels across profiles.

	TOTAL	Profile 0	Profile A	Profile B	Profile C	Profile D	
n	222	85	79	17	30	11	
Prevalence (%)	100%	**38.29%**	**35.59%**	**7.66%**	**13.51%**	**4.96%**	
Male (%)	43.7%	60.0%	38%	82.4%	0%	18.2%	
Mean age (years)	84.58	84.01	82.23	88.59	86.93	93.18	
							
		Mean ± sd ^§^					***p***
					
**Fasting Glucose** (n = 205)	103.88 ± 27.15	105.6 ± 24.6	103.4 ± 31.4	98.81 ± 22.8	103.4 ± 26.3	102.6 ± 25.4	0.919
							
**HbA1c** (n = 155)	6.32 ± 1.04	6.27 ± 0.89	6.49 ± 1.30	6.13 ± 0.82	6.17 ± 0.86	6.33 ± 0.56	0.660
							
**Total Cholesterol** (n = 199)	182.73 ± 37.27	185.5 ± 39.2	178.9 ± 32.8	165.0 ± 36.2	194.7 ± 40.0	182.5 ± 36.7	0.107
							
**HDL-c** (n = 197)	51.63 ± 14.92	52.45 ± 17.6	50.92 ± 12.9	46.26 ± 13.7	51.70 ± 12.0	57.36 ± 12.7	0.416
							
**LDL-c** (n = 197)	109.23 ± 31.64	110.9 ± 32.3	105.6 ± 28.6	97.86 ± 33.5	119.6 ± 35.0	109 ± 29.2	0.202
							
**Triglycerides** (n = 199)	120.61 ± 56.57	123.2 ± 61.5	126.8 ± 60.5	107 ± 37.1	121.5 ± 43.0	80.81 ± 28.3	0.116
							
**Vitamin B12** (n = 143)	357.36 ± 148.29	346.6 ± 135.	369.6 ± 159.	362.7 ± 159.	355 ± 140.	342.6 ± 173.	0.949
							
**Folic acid** (n = 137)	7.44 ± 3.72	7.15 ± 3.08	7.22 ± 4.13	8.17 ± 3.54	7.43 ± 3.59	8.89 ± 4.83	0.654
							
**Vitamin D** (n= 71)	24.49 ± 15.76	26.71 ± 16.7	22.30 ± 13.7	28.05 ± 8.83	21.73 ± 13.5	31.74 ± 28.1	0.654
deficiency ^†^	34 (47.9)	7 (31.8)	18 (54.5)	0 (0.0)	6 (66.7)	3 (60.0)	0.188
							
**Fe TIBC** (n = 158)	331.22 ± 59.17	328.2 ± 58.6	328 ± 54.7	323.9 ± 71.1	354.6 ± 57.6	330.6 ± 70.8	0.454
							
**Creatinine** (n = 203)	1.05 ± 0.47	0.97 ± 0.25	1.08 ± 0.41	1.44 ± 1.19	0.96 ± 0.29	1.13 ± 0.53	**0.008**
							
**Uric acid** (n= 190)	5.76 ± 1.7	5.69 ± 1.64	5.85 ± 1.82	6.21 ± 1.16	5.24 ± 1.33	6.18 ± 2.49	0.350

^§^ Normal range and units of measurement: **Fasting glucose**: 74–100 mg/dL; **HbA1c** 4.7–5.6%, normal; 5.7–6.4%, pre-diabetes; ≥6.5% diabetes. **Total cholesterol** 1–200 mg/dL; **HDL-c** 50–80 mg/dL, normal; <40 ♂, cardiovascular risk; <45 ♀, cardiovascular risk; **LDL-c** 100–130 mg/dL, normal; >130, cardiovascular risk; **triglycerides** 4–150 mg/dL. **Vitamin D** 20–89 ng/mL; **Vitamin B12** 187–883 pg/mL; **Folic acid** 3.1–20 ng/mL; **TIBC** (total iron-binding capacity) 250–450 μg/dL (a high level suggests low iron availability). **Creatinine** 0.72–1.26 mg/dL ♂; 0.57–1.1 mg/dL ♀. **Uric acid** 3.5–7.2 mg/dL ♂; 2.6–6 mg/dL ♀. ^†^ Percentages calculated among tested patients only.

## Data Availability

Data is unavailable due to privacy and ethical restrictions.

## Appendix A

### Appendix A.1. Spanish Adaptation of the Pfeiffer Test

**items**	**respuesta**
	Errónea
**1. ¿Cuál es la fecha de hoy?** (día, mes. año)	
**2. ¿Qué día de la semana?**	
**3. ¿En qué lugar estamos?**	
**4. ¿Cuál es su número de teléfono?** **(si no tiene teléfono ¿Cuál es su dirección completa?)**	
**5. ¿Cuántos años tiene?**	
**6. ¿Cuál es la fecha de su nacimiento?** (día, mes, año)	
**7. ¿Cuál es el nombre del Presidente actual del Gobierno?**	
**8. ¿Cuál es el nombre del Presidente anterior del Gobierno?**	
**9. ¿Cuáles son los dos apellidos de su madre?**	
**10. Reste de tres en tres al número 20 hasta llegar a 0**	
**TOTAL ERRORES**	

**RESULTADOS:**

Valoración cognitiva normal
0–2 errores
Deterioro cognitivo (cognitive impairment)
3–4: Deterioro leve (mild cognitive impairment)
5–7: Deterioro moderado, patológico (moderate cognitive impairment, pathological)
8–10: Deterioro significativo, patológico (significant cognitive impairtment, pathological)
Si no se tiene educación primaria se admite un error más
Si se tienen estudios superiores se admite un error menos.

### Appendix A.2. Spanish Translation of the Barthel Index to Assess Performance in Activities of Daily Living

RESULTADOS: 0–100 puntos (0–90 si en una silla de ruedas).

### Appendix A.3. Questionnaire About Lifestyle

Adherence to the Mediterranean diet was assessed using the questionnaire in Appendix A.4.

## Appendix B

**Table A1:** Fitting parameters for model selection.

npar	LL (df)	AIC	SSBIC	Entropy	BLRT *p* value
10	−1200.653	2421.307	2423.643		
20	−1169.634	2379.267	2383.939	0.741	0
30	−1160.039	2380.079	2387.086	0.75	0.06
**40**	−1143.152	**2366.303**	**2375.647**	**0.844**	**0**
50	−1132.914	**2365.507**	2377.507	0.782	0.048
60	−1126.142	2372.283	2386.299	0.841	0.4

**Table A2:** Conditional probability of cognitive status and autonomy involving age and sex (four-profile model).

	Profile 1	Profile 2	Profile 3	Profile 4
n	135	46	30	11
% of sample	60.81%	20.72%	13.51%	4.96%
gender (% men)	44.9%	70.7%	0%	21.7%
age (mean)	82.16	88.01	86.69	92.90
**BI score ranges**				
0 (independency)	0.479	0.637	**0**	**0**
1 (mild dependency)	0.192	0.155	0.065	0
2 (moderate independency)	0.244	0.113	0.624	0.463
3 (severe dependency)	0.078	0.095	0.273	0.454
4 (total dependency)	0.007	0	0.038	0.083
**Categorized Pfeiffer results**				
0 (normal)	**0.868**	**0.919**	0.434	0.524
1 (mild cognitive impairment)	0.055	0.081	0.347	0.173
2 (moderate cognitive impairment)	0.077	0	0.112	0.302
3 (significant cognitive impairment)	0	0	0.108	0

**Table A3:** Summary of variables with statistical significance.

**Resulting from the descriptive analysis**				
	gender		age	
	*p*			*p*
Cognitive status	0.000		Cognitive status	0.024
Autonomy	0.000		Autonomy	0.023
Lifestyle				
Education	0.005		Hearing loss	0.002
Physical activity	0.000		Physical activity	0.052 ^§^
Smoking	0.000			
Alcohol consumption	0.004			
Chronic health conditions				
CVD	0.060 ^§^		COPD	0.046
COPD	0.001		Cataracts	0.035
Osteoarticular	0.000			
Depression	0.000			
Insomnia	0.002			
Anxiety	0.003			
Comorbidity	0.019			
Blood biochemical parameters				
Total Cholesterol (n = 199)	0.000		HbA1c (negative correlation)	0.056
HDL-c (n = 197)	0.000		Triglycerides (negative correlation)	0.006
LDL-c (n = 197)	0.001		Altered TIBC	0.022
Creatinine (n = 203)	0.001			
Uric acid (n = 190)	0.000			
B12	0.008			
TIBC	0.010			
**Resulting from the inter-profile analysis**				
Lifestyle				
Physically active	0.000			
Never smoking	0.001			
Chronic health conditions				
CKD	0.046			
COPD	0.050			
Osteoarticular	0.017			
Depression	0.067 ^§^			
Anxiety	0.021			
Comorbidity	0.002			
Blood biochemical parameters				
Creatinine	0.008			

^§^ Approaching statistical significance.
